# Possible implications of an accessory abductor digiti minimi muscle: a case report

**DOI:** 10.1186/1749-7221-2-22

**Published:** 2007-12-03

**Authors:** Luis Ernesto Ballesteros, Luis Miguel Ramirez

**Affiliations:** 1 Basic Science Department, Medicine Faculty, Universidad Industrial de Santander, Bucaramanga, Colombia; 2 Department of Basic Science, Medicine Faculty, Universidad Industrial de Santander, Bucaramanga, Colombia

## Abstract

**Background:**

Accessory ADM was first reported in 1868 although muscular, vascular and nervous variations of the hypothenar eminence are rare, contrary to anomalous muscles in the wrist which are relatively common.

**Case presentation:**

This case report presents a bilateral variation of an accessory abductor digiti minimi muscle in a male specimen. Ulnar artery and ulnar nerves were taken into account regarding their position and trajectory related to this variation.

**Conclusion:**

Muscle size may be an important factor in considering whether a variation is able to produce neurovascular compression and clinical implications.

## Background

This case report presents an abductor digiti minimi (ADM) muscle's bilateral accessory head, involving ulnar artery (UA) and ulnar nerve (UN) trajectories' sensorial and motor components. Although muscular, vascular and nervous variations of the hypothenar eminence are rare (differing from anomalous muscles in the wrist), they have been reported [1-10]. According to Sheppard, three cases of accessory ADM were first reported by Wood in 1868. Most authors call such additional ADM head an "accessory" component which can be unilaterally or bilaterally presented, producing compression in Guyon's canal. Harvie *et al*., found an accessory ADM in 41% of 116 volunteers' asymptomatic ultrasound examinations, greater prevalence being found in male samples and bilateral presentation in 50% of cases for both genders.

The flexor digiti minimi brevis muscle can also be present in this area; Madhavi *et al*., have stated that it relates to the common phylogeny of these muscles from the same muscle mass [11]. Soldado-Carrera *et al*., found this variation to be associated with decreased flexor digitorum superficialis muscle fourth tendon caliper and median nerve split. Murata [7] found an accessory ADM in 35 hands, having one (17%), two (80%) and three fascicles (3%). Furthermore, Wulle [8] presented eleven cases of accessory ADM, but having "longus" presentation.

## Case presentation

**Figure 1:**
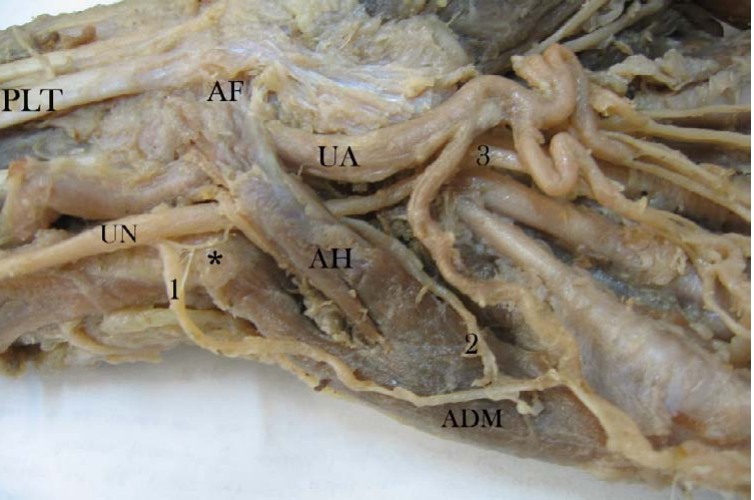
Left-hand hypothenar area. ADM: abductor digiti minimi muscle. AH: accessory head of abductor digiti minimi muscle. UA: ulnar artery. UN: ulnar nerve. AF: antebrachial fascia. PLT: palmaris longus tendon muscle. Asterisk (*): pisiform bone. 1: Dorsal cutaneous branch of ulnar nerve. 2: UN deep sensory branch trajectory. 3: Fourth common digital nerve.

**Figure 2:**
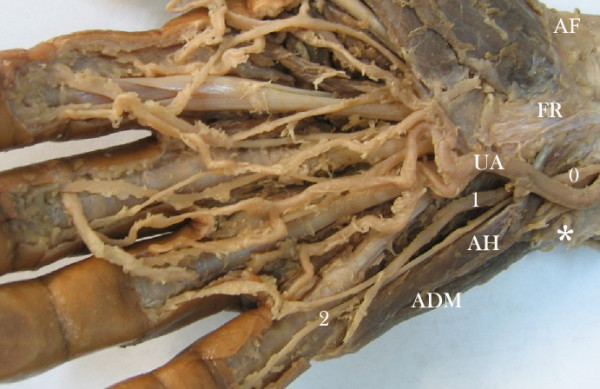
Right-hand hypothenar zone. ADM: abductor digiti minimi muscle. AH: accessory head of abductor digiti minimi muscle. UA: ulnar artery. FR: flexor retinaculum. AF: antebrachial fascia. Asterisk (*): pisiform bone. 1, 2: ulnar nerve sensorial digital branches. 0: ulnar nerve.

This case report arises from one cadaver specimen amongst a sample of thirty which were obtained from Universidad Industrial de Santander's Medical Faculty's Anatomy Department (dissection laboratory) during 2006 academic semesters. The specimen was fixed in 10% formaldehyde solution. Dissections were performed by the authors, involving the antebrachial and hand dorsal and ventral area. The hypothenar area was carefully dissected following vascular and nervous structures neighbouring the ADM's accessory head. These findings were recorded and photographed (digital camera and an electronic Mitutoyo calliper) according to UN side and collateral origin, UA vascular distribution and muscular variation. The left hand (Figure 1) presented an accessory ADM head originating in antebrachial fascia and palmaris longus tendon. According to Murata *et al*., [7] classification resembles "variation 2", however without additional palmaris longus tendon origin. It passed obliquely through Guyon's canal, enclosing the UN and vessels. The accessory head had a late union in its distal trajectory to form a single pennate with the ADM originating in pisiform bone. It was localized over the flexor digiti minimi brevis muscle and lateral to opponens digiti minimi muscle. Left variation length was 50.6 mm, 11.65 mm wide and 2.43 mm thick compared to the ADM ipsilateral usual head which was 55.9 mm, 13.03 mm wide and 7.09 mm thick. This left accessory ADM head had 34.3% of the thickness of a usual ADM head. UN and UA were covered by this ADM accessory head. Sensorial UN branches had both superficial and deep trajectories regarding muscular variation and were presented in relation to their trajectory as superficial sensory branch trajectory coming from the ulnar nerve's dorsal cutaneous branch and a deep sensory branch trajectory coming from the ulnar trunk. These abnormal ulnar nerve branches fitted Kaplan's variation type 4 according to Hankins et al. [12]. Kaplan's accessory branch (considered a rare anatomic variant) together with accessory ADM in the same case have never been reported in the literature. They were connected after coursing the accessory ADM head. A third sensorial trajectory course was observed toward the fourth finger after passing Guyon's canal as the "fourth common digital nerve". The right hand (Figure 2) also presented an accessory ADM head originating in the flexor retinaculum and palmaris longus tendon. According to Murata *et al*., [7] classification resembles "variation 1" however without additional palmaris longus tendon origin. It also passed obliquely through Guyon's canal, enclosing the UN. This accessory head also had a late union in its distal trajectory to form a single pennate with the ADM originating in pisiform bone. It was also localized over the flexor digiti minimi brevis muscle and lateral to opponens digiti minimi muscle. Right hand variation length was 37.64 mm, 8.59 mm wide and 3.71 mm thick compared to the usual ipsilateral ADM head which was 57.7 mm, 13.1 mm wide and 6.92 mm thick. This accessory ADM head had 45.8% of the thickness of a usual ADM head. The UA adopted a superficial course above the accessory ADM head while the UN and all its sensorial branches were covered by this variation. According to UN arborisation patterns by Murata *et al*., [7] it looked like a "type 4" classification. These accessory ADM heads received deep UN branch motor innervations on both sides.

This class of muscular variation entrapping neuro-vascular bundles may have sensorial and/or muscular implications in a range of compression neuropathies [5,6]. Entrapment neuropathies can produce heterotopic projected pain, symptomatic consequences arising from mechanical nerve injury passing through a narrow anatomical space or under a muscular structure which may potentially compress the neuro-vascular package [13-15]. We think that muscle size, tightness and course may be important factors in considering whether such variations are able to produce compression.

This scenario can cause nerve oedema and ischemia by muscle structure friction over the peryneuro which can cause neuritis and raise endoneural pressure. Involvement of vegetative contributions must be taken into account when assessing the effects of such sensorial injuries, due to their ability to produce complex pain syndromes [16,17]. In addition to traumatic neuropathic pain, symptoms in distal nerve distribution (dysesthesia, paresthesia and anaesthesia), paresis, hyporeflex, hypotonicity and atrophy, such as inferior motor neuron lesion, may be equally feasible [18,19]. The deep UN motor branch may be compressed by this ADM accessory head and compromise palmar and dorsal interosseous muscles, hypothenar eminence muscles, lumbricals III and IV and hallux adductor, thereby producing a clinical "claw hand" appearance or resembling Guyon's canal syndrome.

Having dealt with this variation's possible sensorial and motor effects, vascular effects must also be considered. UA compression (left hand) may produce peripheral vascular disease from two possible events depending on the type of compression (i.e. constant or intermittent muscular compression). Muscular hyperfunction can reduce these arterial branches' distal irrigation, causing hypoesthesia or hyperesthesia in the hand. Likewise, muscular spasm can produce anaerobic metabolism and consequent nerve irritation by anoxic acidified setting with neurogenic inflammation and peripheral hyperalgesia [20,21]. Permanent loss of vascular supply during muscular spasm can lead to vascular claudication and pain [22,23].

We believe (from a functional perspective) that precipitating factors (gender, occupation, side dominance, traumatic history, anatomical characteristics) may develop neurovascular compression and symptoms in an anomalous muscle; we thus present this anatomical case. The morphological characteristics led to all the above explained symptomatic possibilities; however, lacking clinical judgment, further statements lose their functional value and become merely speculative.

## Conclusion

Muscular variations compromising neurovascular bundles may produce clinical symptoms such as dysaesthesic pain, sensory loss, wakefulness and paresis, highlighting these anatomical discrepancies' importance when they are presented. However, the existence of an accessory ADM is usually asymptomatic and only rarely results in nerve compression. It does appear that muscle size, tightness and course may be an important factor in considering whether such variation is able to produce UN compression at Guyon's canal or UA compression resulting in a poor relationship between blood-flow and artery diameter. A larger sample of cadaver specimens is needed. Also, further clinical studies are required to confirm the existence of these variations' clinical effects.

## List of abbreviations

Abductor digiti minimi muscle – ADM

Ulnar nerve – UN

Ulnar artery – UA

## Authors' contributions

Luis Ernesto Ballesteros Acuña – ES, FG

Luis Miguel Ramirez Aristeguieta – ES, FG
